# Doping-free complementary WSe_2_ circuit via van der Waals metal integration

**DOI:** 10.1038/s41467-020-15776-x

**Published:** 2020-04-20

**Authors:** Lingan Kong, Xiaodong Zhang, Quanyang Tao, Mingliang Zhang, Weiqi Dang, Zhiwei Li, Liping Feng, Lei Liao, Xiangfeng Duan, Yuan Liu

**Affiliations:** 1 Key Laboratory for Micro-Nano Optoelectronic Devices of Ministry of Education, School of Physics and Electronics, Hunan University, 410082 Changsha, China; 2 State Key Lab of Solidification Processing, College of Materials Science and Engineering, Northwestern Polytechnical University, 710072 Xi’an, China; 3 State Key Laboratory for Chemo/Biosensing and Chemometrics, College of Chemistry and Chemical Engineering, Hunan University, 410082 Changsha, China; 4 Department of Chemistry and Biochemistry, University of California, Los Angeles, CA 90095 USA

**Keywords:** Electronic properties and materials, Electronic devices

## Abstract

Two-dimensional (2D) semiconductors have attracted considerable attention for the development of ultra-thin body transistors. However, the polarity control of 2D transistors and the achievement of complementary logic functions remain critical challenges. Here, we report a doping-free strategy to modulate the polarity of WSe_2_ transistors using same contact metal but different integration methods. By applying low-energy van der Waals integration of Au electrodes, we observed robust and optimized p-type transistor behavior, which is in great contrast to the transistors fabricated on the same WSe_2_ flake using conventional deposited Au contacts with pronounced n-type characteristics. With the ability to switch majority carrier type and to achieve optimized contact for both electrons and holes, a doping-free logic inverter is demonstrated with higher voltage gain of 340, at the bias voltage of 5.5 V. Furthermore, the simple polarity control strategy is extended for realizing more complex logic functions such as NAND and NOR.

## Abstract (web summary)

One of the challenges hindering the control of 2D transistor polarity is the incompatibility with conventional ion-implantation doping approaches. Here, the authors report a doping-free strategy to obtain polarity control of WSe_2_ transistors using same-metal contacts with different integration methods.

## Introduction

Two-dimensional (2D) semiconductors have attracted considerable attention as ultrathin channel materials for transistors^1–5^. Their atomically thin body and dangling-bond free surface offer significant potential for ultimate transistor scaling (down to atomic thin-body thickness), which is essential for decreasing off-state power consumption and further extending Moore’s Law^6^. To date, one major challenge of a 2D transistor is the uncontrollable device polarity (n- or p-type) and majority carrier type, posing a key limitation for realizing complementary metal oxide semiconductor (CMOS) logic function in 2D transistors. In modern silicon microelectronics, the doping concentration of the silicon channel and transistor polarity are achieved by introducing extrinsic (e.g., B for p-type and As for n-type) dopants through high-energy ion implantation and subsequently high-temperature activation^7,8^. However, applying existing state-of-the-art ion-implantation approaches to a 2D semiconductor is not straightforward because there is little physical space for impurity dopants in such atomically thin lattice^9^. Hence, the majority carrier type of a typical 2D transistor is limited to its intrinsic properties and is largely fixed once exfoliated or synthesized.

Considerable efforts have been devoted to realize 2D CMOS functions in the past few years^10–13^. Early attempts focused on using two different 2D semiconductors, where one material is used for the NMOS (e.g., MoS_2_ and MoSe_2_) and a different material is used for PMOS (e.g., black phosphorus, WSe_2_)^14–16^. Although demonstrating desired logic functions, this method is still relied on the uncontrollable intrinsic doping, and is not obviously compatible with CMOS technology since it involves two materials with distinct synthesizing and processing conditions. Alternatively, the selective doping of 2D semiconductors can be achieved through a gentle chemical surface absorption with charge transfer process between 2D semiconductors and adsorbate, which could effectively modulate 2D carrier concentration and their majority carrier type (electrons or holes). For example, polyethyleneimine or benzyl viologen molecule was employed to achieve n-type doping in multilayer MoS_2_ (refs. ^17,18^), and the chloride molecule was applied to increase electron-doping density of WS_2_ and MoS_2_ (ref. ^19^). However, such chemical absorption approaches typically suffer from poor stability due to the weak interaction between the surface dopants and 2D materials. Recently, the CMOS logic functions are also demonstrated in 2D channels (e.g., WSe_2_ and MoTe_2_) using metals with different work function. For example, Ag and Pt have been applied as the contact metal of WSe_2_ to achieve the NMOS and PMOS, respectively, and similarly, Ti and Pt are integrated in MoTe_2_ flake to realize CMOS inverter^20,21^. However, due to the strong Fermi level pinning effect, large Schottky barrier is typically observed in 2D/metal interfaces, regardless of the metal work function used^22–25^. Therefore, using this approach, it is difficult to achieve optimized device performance in both p- and n-type devices at the same time. In addition, the use of asymmetric contact metals could further complicate the fabrication processes.

Here, we report a doping-free strategy to achieve CMOS circuit functions by using the same contact metal gold (Au) and the same channel material WSe_2_, but different metal integration methods. By applying low-energy van der Waals (vdW) integration of Au electrode, we observed a robust and consistent p-type behavior in multilayer WSe_2_. This is in great contrast to the transistors fabricated on the same WSe_2_ flake using conventional deposited Au contacts, where pronounced n-type characteristic is always observed^26,27^. To further gain insight of this phenomenon, we conducted detailed analysis through thickness-dependent measurement and density functional theory (DFT) simulation, and attributed the polarity change of WSe_2_ to the controllable Fermi level pinning effect using different metal integration methods. With the ability to control the polarity of WSe_2_ transistors and to achieve optimized contact to both PMOS and NMOS using the same metal, a logic inverter is demonstrated with the highest voltage gain of 340 (at a bias voltage of 5.5 V) and total noise margin over 90%. Furthermore, the polarity-controllable strategy is also extended to realize more complex logic functions such as NAND and NOR. Our results not only demonstrate robust and high-performance CMOS logic circuit using vdW metal electrodes, but also provide a doping-free method to control the polarity of a 2D semiconductor using the same contact metal, shedding light to high-performance 2D electronics and CMOS design.

## Results

### Fabrication processes and electrical measurement

**Fig. 1:**
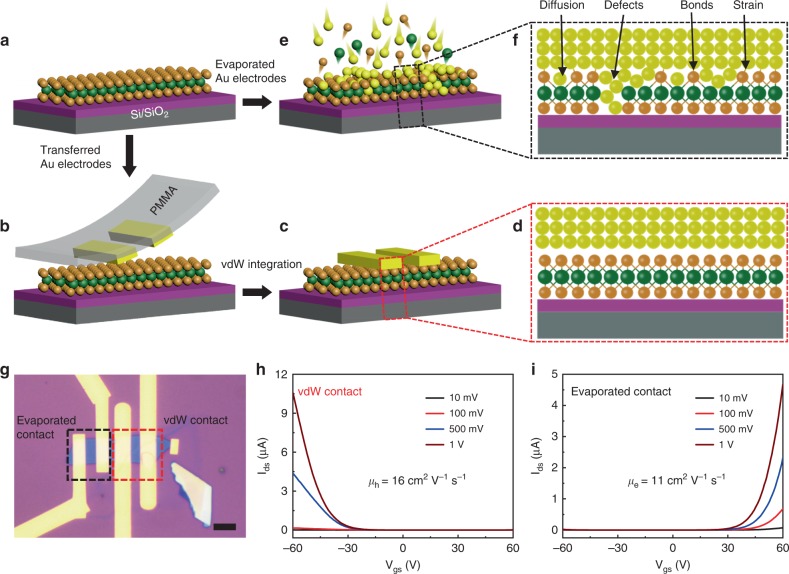
Schematical illustration of device structure and electrical measurement. **a**–**c** Fabrication processes of WSe_2_ transistors using vdW integration processes: WSe_2_ flake exfoliated onto Si/SiO_2_ substrate (**a**); pre-fabricated Au electrodes physically laminated on WSe_2_ surface with weak vdW interaction (**b**, **c**). **d** The cross-sectional schematic of vdW contact with WSe_2_, demonstrating clean and sharp interfaces. **e** Au electrode evaporated on WSe_2_ using conventional thermal evaporation. **f** The cross-sectional schematics of evaporated contact with WSe_2_, demonstrating a highly disordered interface. **g** Optical image of a typical fabricated device with both evaporated (left pair, highlighted by a black box) and vdW-integrated (right pair, highlighted by a red box) electrodes on the same WSe_2_ flake (~7-nm thick). The scale bar is 5 μm. **h**, **i** The *I*_ds_−*V*_gs_ transfer characteristics of the WSe_2_ transistor (shown in **g**) using both vdW-integrated (**h**) and conventional evaporated electrodes (**i**). By controlling the metal integration approaches, the device polarity can be switched between p- and n-type, with a carrier mobility of 16 and 11 cm^2^ V^−1^ s^−1^ (at a bias of 1 V), respectively.

Figure 1a–f schematically illustrates our device structure. To fabricate the device, multilayer WSe_2_ flakes with various thicknesses are first mechanically exfoliated onto a heavily doped silicon substrate (as gate) with 300-nm silicon oxide (as gate dielectric). Next, 50-nm Au electrode pair is pre-fabricated on a sacrificial Si wafer and then mechanically released using a previously developed method^26^ (see “Methods” section for fabrication details). The released metal electrodes are aligned under a microscope and physically laminated on top of the WSe_2_ flake using a vdW metal integration process, resulting in an atomically sharp and clean Au/WSe_2_ interface^26,27^ (Fig. 1a–d). For comparison, another pair of Au electrode with the same thickness (50-nm thick) is also deposited on the same WSe_2_ flake using conventional electron beam lithography followed by high vacuum thermal deposition, resulting in the nonideal metal/semiconductor interfaces with diffusion, defects, chemical bonding, and strains, as have been demonstrated previously^26,27^ and schematically illustrated in Fig. 1e, f. The optical image of a typical fabricated device is shown Fig. 1g, where the left electrode pair is fabricated through thermal evaporation (highlighted by a black box) and the right electrode pair (red box) is vdW integrated. Electrical transport studies of the resulting devices were carried out at room temperature in a probe station under vacuum condition (3 × 10^−5^ Torr). As shown in Fig. 1h, a typical device (~7-nm thick) contacted with vdW metal electrodes shows p-type *I*_ds_−*V*_gs_ transfer characteristic, consistent with band alignment of WSe_2_ with high work function Au, suggesting the optimized Au/WSe_2_ interface using the vdW metal integration approach^28–30^. In contrast, without applying any doping process, n-type *I*_ds_−*V*_gs_ transfer characteristic is observed in the control device (fabricated on the same WSe_2_ flake) using conventional deposited Au contacts (Fig. 1i). The observed polarity change indicates the strong Fermi level pinning effect within evaporated Au/WSe_2_ interfaces, where the pinned Fermi level position is close to the conduction band of WSe_2_. Furthermore, the two-terminal FET mobility *μ* can be further extracted using equation *μ* = [d*I*_ds_/d*V*_gs_] × [*L*/(*WCV*_ds_)], where *L*/*W* is the ratio between channel length and width (shown in Fig. 1g), *C* is the back-gate capacitance (1.15 × 10^−8^ F cm^−2^, 300-nm-thick SiO_2_). The extracted hole and electron mobility in this device are 16 and 11 cm^2^ V^−1^ s^−1^, respectively. In addition, the contact resistance (*R*_c_) and Schottky barrier height (SBH) of both p- and n-type transistors can also be extracted using the transfer line method and temperature-dependent measurement, where *R*_c_ and SBH are measured to be 14 kΩ μm, 50 meV for PMOS and 17 kΩ μm, 60 meV for NMOS, respectively, as shown in Supplementary Fig. 1. The balanced *μ*, *R*_c_, and SBH between electrons and holes are important for the demonstration of high-performance CMOS circuit described below.

### Thickness-dependent electrical measurement

**Fig. 2:**
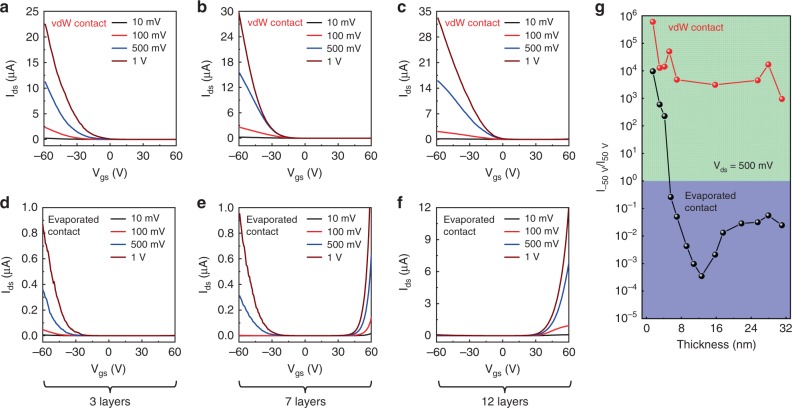
Thickness-dependent electrical measurement of WSe_2_ transistors with vdW-integrated and evaporated Au electrodes. **a**–**c**, *I*_ds_−*V*_gs_ transfer curves of WSe_2_ with different thicknesses (3 layers in **a**, 7 layers in **b**, and 12 layers in **c**) using vdW Au electrodes, where p-type behavior is consistently observed. **d**–**f** *I*_ds_−*V*_gs_ transfer curves of WSe_2_ with different thicknesses (3 layers in **d**, 7 layers in **e**, and 12 layers in **f**) using conventional deposited Au electrodes, where p-type, bipolar, and n-type behaviors are observed in **d**, **e**, and **f**, respectively. The *V*_ds_ bias voltage is 0.01 V (black), 0.1 V (red), 0.5 V (blue), and 1 V (brown) throughout **a**–**f**. **g** The current ratio between *I*_−50V_ (*I*_ds_ at *V*_g_ = −50 V) and *I*_50V_ (*I*_ds_ at *V*_g_ = 50 V) as a function of WSe_2_ thickness. For devices with vdW electrodes, large *I*_−50V_/*I*_50V_ ratio >10^3^ is observed, suggesting the consistent p-type behavior, regardless of the channel thickness. For devices with conventional evaporated Au electrodes, *I*_−50V_/*I*_50V_ is decreased with increasing body thickness, where a p-type to n-type transition is clearly observed. The *V*_ds_ bias voltage in **g** is fixed at 500 mV.

To further confirm the robustness of this behavior and investigate the polarity control by using different metal integration processes, we have conducted detailed electrical measurement based on WSe_2_ of various thicknesses. As shown in Fig. 2a–c, the device contacted with vdW metal electrodes shows clearly p-type behavior, regardless of the WSe_2_ thickness, in consistent with the band alignment between WSe_2_ valance band (5.02–4.83 eV from monolayer to bulk) and high work function Au (5.24 eV). In great contrast, the control devices (contacted with conventional evaporated Au electrodes) display a unique polarity change behavior with increasing WSe_2_ thickness, demonstrating p-type characteristic with thickness <5 layers (~3 nm), a bipolar characteristic with 7 layers thick (~4.5 nm), and pronounced n-type property with thickness greater than 10 layers (~6.5 nm), as shown in Fig. 2d–f. The corresponding on–off ratio and mobility of these transistors (in Fig. 2a–f) and monolayer WSe_2_ transistor data (using both metal integration processes) are also plotted in Supplementary Fig. 2.

Furthermore, to confirm the robustness of this behavior and to quantitively analyze the polarity change, we have measured over 20 devices and extracted the current ratio between *I*_−50V_ (*I*_ds_ at *V*_g_ = −50 V) and *I*_50V_ (*I*_ds_ at *V*_g_ = 50 V) as a function of WSe_2_ thickness. The *I*_−50V_/*I*_50V_ ratio here could represent the ratio between hole and electron contribution in a given transistor, and thus could quantitively demonstrate the transistor polarity and majority carrier type. For devices with vdW-contacted electrode (Fig. 2g, red dot), the *I*_−50V_/*I*_50V_ ratio over 10^3^ is consistently observed from monolayer to 30-nm- (~50 layers) thick devices, suggesting a dominated p-type behavior (with negligible electron current) regardless of body thickness. In contrast, for devices with conventional evaporated electrodes, the *I*_−50V_/*I*_50V_ ratio decreased exponentially from 10^4^ to ~10^−3^ (7 orders of magnitudes) with increasing body thickness from monolayer to ~50 layers, demonstrating that the majority carrier type can be progressively transformed from holes to electrons via increasing the thickness of WSe_2_. The slightly increased *I*_−50V_/*I*_50V_ ratio for evaporated contacts (with thickness >13 nm, black line of Fig. 2g) could be attributed to the increased vertical resistance (under contact region) with increasing body thickness.

Moreover, the devices integrated by both vdW and evaporated electrodes are very stable, which can exhibit the original device polarity after 4 months of storage at room temperature in ambient atmosphere (Supplementary Fig. 3), further suggesting the stability of our doping-free approaches^25,26^. We also note that the unique device polarity control technique reported here is not only limited to WSe_2_ and Au metal, but could be extended to other 2D semiconductor–metal systems by using different contact integration processes to pin (using evaporated contact) or de-pin (using vdW contact) the Fermi level, as demonstrated in a MoS_2_–Pt system in Supplementary Fig. 4.

### DFT simulation

**Fig. 3:**
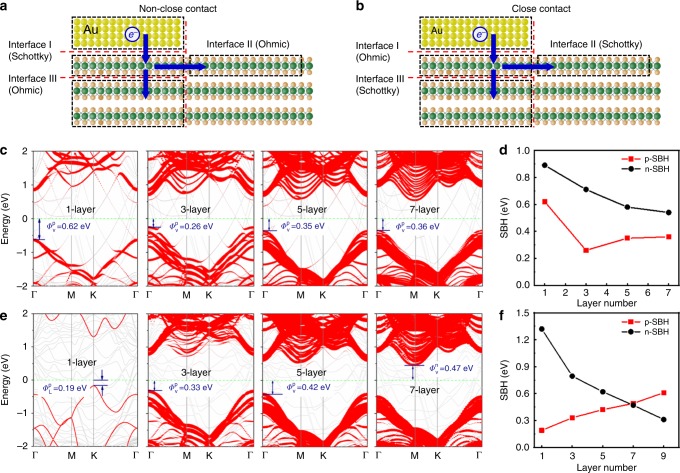
DFT calculation of Au/WSe_2_ interface with different contact approaches. **a**, **b** Schematic cross-sectional view of the Au/WSe_2_ non-close-contact model (**a**) and the close-contact model (**b**). **c** Calculated band structures of WSe_2_ with different thickness (under Au contact) for the non-close-contact model. The red dots denote the projected band structures of WSe_2_ underlying the Au electrode, and the dots size represents the weights. **d** Variation of calculated SBH with WSe_2_ layer number for the non-close-contact model, where dominated p-type SBH is always observed. **e** Calculated band structures of different thickness of WSe_2_ under Au for the close-contact model. The red dots denote the projected band structures of WSe_2_ underlying the first layer WSe_2_, and the dot size represents the weights. **f** Variation of calculated SBH with WSe_2_ layer number for the close-contact model, with a clear transition from p- to n-type SBH with increasing layer thickness.

To further understand the mechanism of polarity control by using different metal integration approaches, and to gain insight into the thickness dependent on PMOS to NMOS transition, we have carried out DFT simulation of carrier transport across the metal/WSe_2_ interfaces. First, we constructed two types of Au/WSe_2_ interface models, a close-contact model corresponding to the evaporated Au interface and a non-close contact corresponding to the vdW-integrated Au interface. For the close-contact model, an interlayer distance of 1.5 Å (covalent radius of Au and Se) was chosen between metal and WSe_2_, under which the Au and the Se atoms are covalently bonded. For the non-close-contact model, an interlayer distance of 3.3 Å was used, which included an additional vdW-gap distance of 1.8 Å on the base of close-contact interlayer distance, consistent with previous reports^9^. Based on this model, there are three interfaces that may contribute to the transport barrier: Au and the first layer WSe_2_ (interface I), WSe_2_ under the contact and inside the channel region (interface II), as well as the first layer WSe_2_ and the rest of the WSe_2_ layers (interface III), as illustrated in Fig. 3a, b.

For the non-close contact, as the Au/WSe_2_ interlayer distance is large enough and their interlayer interaction is weak, Au electrode has little influence on the properties of WSe_2_. As shown in Supplementary Fig. 5, there are negligible interfacial gap states in WSe_2_, and the whole multilayer WSe_2_ maintains its intrinsic properties, leading to Ohmic contacts at interfaces II and III. Therefore, the contact Schottky barrier only exists in interface I, regardless of the thickness of WSe_2_ used. Figure 3c illustrates the calculated band structures of WSe_2_ under vdW Au contact, which is nearly the same with that of freestanding WSe_2_ (Supplementary Fig. 6), further indicating the weak interaction between Au electrode and the underlying WSe_2_. The calculated results of SBH are shown in Fig. 3d with dominating p-type Schottky barrier, consistent with observed p-type transistor behavior using vdW Au contact (Fig. 2a–c).

In great contrast, for evaporated Au with the close-contact model, chemical interaction exists between Au electrode and WSe_2_, which strongly perturbs the electrical properties of WSe_2_. As shown in Supplementary Fig. 7, a large number of interfacial states are generated in the forbidden band of WSe_2_, resulting in the disappearance of the WSe_2_ bandgap. Therefore, as demonstrated in Fig. 3b, the first layer of WSe_2_ is metalized under the contact (with a new work function ~4.83 eV), leading to an Ohmic contact at interface I. Meanwhile, Schottky barrier is generated at interfaces II and III during charge transport from metalized WSe_2_ (under contact) to semiconducting WSe_2_. For monolayer WSe_2_, the lateral Schottky barrier at interface II is p-type with a barrier height of 0.19 eV, as revealed by the calculated band alignments (Supplementary Fig. 8). On the other hand, for multilayer WSe_2_, the first underlying WSe_2_ is metalized, but the rest of the underlying layers remains largely intrinsic (Supplementary Fig. 9), and consequently the effect of Schottky barrier at interface III is more and more pronounced. As shown in Fig. 3e, f, the calculated vertical Schottky barriers at interface III are p-type when Au electrode contacts with 3-layer and 5-layer WSe_2_, and gradually switched to n-type with 7-layer and 9-layer WSe_2_, consistent with our measurement results in Fig. 2d–g. Figure 3f demonstrates the variation of SBH at interface III with layer number, with a detailed mechanism in Supplementary Fig. 10.

### WSe_2_-based CMOS logic functions

**Fig. 4:**
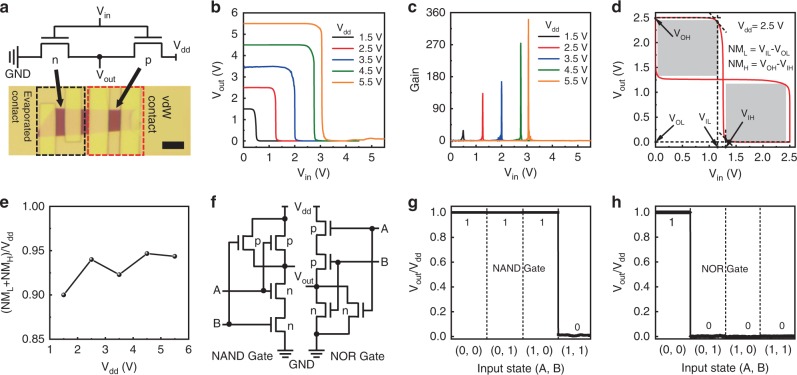
CMOS logic functions based on WSe_2_ transistors with different contact approaches. **a** Circuit diagram (upper) and optical image of a typical complementary inverter composed of two WSe_2_ transistors in series, where one is contacted with deposited Au electrodes (n-type) and another is contacted by vdW Au electrodes (p-type). Scale bar in the optical image is 4 μm. **b** The voltage transfer characteristics of the inverter as a function of the input voltage with different *V*_dd_ from 1.5 to 5.5 V (1-V step). **c** The corresponding voltage gains of the resulting inverter. **d** The bistable hysteresis voltage transfer characteristics of WSe_2_ CMOS logic inverter as a function of the input voltage (*V*_dd_ = 2.5 V), with the noise margin low (NM_L_) of 1.16 V and noise margin high (NM_H_) of 1.19 V achieved. The *V*_OH_, *V*_OL_, *V*_IL_, and *V*_IH_ represent the minimum high output voltage, maximum low output voltage, maximum low input voltage, and minimum high input voltage for the inverter, respectively. **e** The ratio of the total noise margin as a function of *V*_dd_. **f** NAND and NOR circuit diagram composed of four WSe_2_ transistors, where two are contacted with deposited Au electrodes as n-type devices, and another two are contacted by vdW electrodes (p-type). **g**, **h** The input–output logic functions of NAND (**g**) and NOR (**h**) circuits. Gate voltage of −30 and 0 V is used as input “0” and “1”, respectively. *V*_ds_ bias voltage is fixed at 0.23 V.

The ability to control the transistor polarity can readily allow us to integrate multiple WSe_2_ transistors into functional circuits. For example, a complementary logic inverter can be achieved by connecting two WSe_2_ transistors in series, where one device is connected with deposited Au electrodes as n-type transistor and the other is contacted by vdW electrodes as a p-type device. The logic diagram and optical image of the inverter are shown in Fig. 4a, where the metal integration processes (both evaporation and vdW integrated) are the same as previous devices in Fig. 1, except that the back-gate dielectric is changed from 300-nm-thick SiO_2_ to 20-nm-thick Al_2_O_3_ to enhance the gate capacitance and electrostatic control over the channel, which is essential to reduce the inverter input voltage and to increase the voltage gain. The detailed inverter fabrication process is shown in the “Methods” section and Supplementary Fig. 11.

Figure 4b shows the voltage transfer characteristics of the resulting inverter as a function of input voltage with bias voltage (*V*_dd_) from 1.5 to 5.5 V, demonstrating sharp voltage transition with input voltage. The resulted voltage gain is plotted in Fig. 4c with a peak value of 340 at *V*_dd_ = 5.5 V. To the best of our knowledge, the voltage gain reported here represents the highest value for TMD-based inverter, as shown in the comparison with previous literatures in Supplementary Table 1. Further increasing the *V*_dd_ leads to much increased gate leakage current, and degrades the overall device performance. The much higher voltage gain achieved here could be largely attributed to the optimized contact for both PMOS and NMOS by controlling their Fermi level position, which is intrinsically different compared with previous methods by evaporating metals with different work functions, where optimized contact to both PMOS and NMOS is hard to realize due to strong Fermi level pinning effect at metal/2D interfaces^22–25^. To characterize the robustness of an inverter fabricated through different contact approaches, we have extracted the noise margins (NM_L_ and NM_H_), as shown in Fig. 4d. At the *V*_dd_ of 2.5 V, NM_L_ of 1.16 V and NM_H_ of 1.19 V are extracted. In addition, we also plot the total noise margin [(NM_L_ + NM_H_)/*V*_dd_] as a function of *V*_dd_ from 1.5 to 5.5 V (Fig. 4e). The measured total noise margin of the inverter is greater than 90% at various bias voltages, indicating the high tolerance to noise. Furthermore, the static peak energy consumption of the corresponding inverter is also plotted in Supplementary Fig. 12.

Taking a step further, more complicated logic functions could be achieved by connecting more WSe_2_ transistors together. For example, a logic NOR or NAND function can be created using four multilayer WSe_2_ transistors, with two transistors using vdW Au contacts (p-type) and other two using evaporated Au contacts (n-type), as shown in the circuit diagram in Fig. 4f. The measured input and output voltages clearly demonstrate the desired logic function for the NOR and NAND (Fig. 4g, h), suggesting its potential for a more complex circuit.

## Discussion

In summary, we have demonstrated a doping-free strategy to control the polarity of 2D transistors using the same contact metal Au and the same channel material WSe_2_, but different metal integration methods. Through detailed thickness-dependent measurement and DFT calculation, we found that the unique polarity change could be attributed to the controllable Fermi level pinning (or de-pinning) effect using different metal integration methods. Furthermore, with optimized contact to both PMOS and NMOS, we demonstrate a logic inverter with the highest voltage gain of 340 (at *V*_dd_ of 5.5 V) and the total noise margin over 90%, as well as more complex CMOS functions such as NAND and NOR. Our results not only demonstrate high-performance CMOS logic circuit, but also provide a method to control the polarity of a 2D semiconductor using the same contact metal, shedding light to high-performance 2D electronics and CMOS design.

## Methods

### Fabrication process of metal electrode for vdW integration

First, we prepared 50-nm-thick Au electrode arrays on sacrificial silicon substrate (with an atomically flat surface) using standard photolithography followed by thermal evaporation under vacuum (pressure ~5 × 10^−^^4^ Pa). After the lift-off process, the whole wafer was immersed in a sealed hexamethyldisilazane (HMDS) chamber to functionalize the surface of SiO_2_ at 80 °C. Next, the poly(methyl methacrylate) (PMMA A8, Mircochem Inc.) layer was spin-coated twice on the substrate with a speed of 3500 r.p.m. Finally, the 1-μm-thick PMMA layer with array Au electrodes is mechanically peeled and laminated to the target substrate via the mechanical aligner under an optical microscope^26^.

### Inverter fabrication process

For fabricating logic inverter, we first prepared 10/50-nm-thick Ti/Au electrode onto an Si/SiO_2_ substrate as back-gate electrode. Next, the growth of a 20-nm-thick Al_2_O_3_ dielectric layer was employed through atomic layer deposition (ALD) on the gate electrode at the growth temperature of 150 °C. By contacting with vdW and evaporated electrode pairs, PMOS and NMOS devices can be achieved, as shown in Supplementary Fig. 11.

### DFT computational methods

All the calculations were performed based on the DFT in conjunction with projector-augmented wave potentials, which is implemented in the Vienna ab initio Simulation Package (VASP)^31,32^. The generalized gradient approximation in the Perdew, Burke, and Ernzerhof (GGA–PBE) was used to describe the exchange and correlation potential^33^ as PBE bandgap is a good approximation for the transport gap in an FET, and accordingly the SBHs calculated by PBE are closer to the experimental value^34,35^. vdW interaction is taken into account by the DFT-D3 approach^36^, and the energy cutoff for plane waves was set at 450 eV. Geometry optimizations were terminated when the total energy and atomic force are less than 10^−^^5^ and 0.02 eV Å^−^^1^, respectively. A Monkhorst–Pack k-point mesh of 9 × 9 × 1 was used for the calculation of Au/WSe_2_ interfaces. To avoid the interaction effect of adjacent slabs, the thickness of vacuum region was set to no less than 15 Å. The $\sqrt 3 \times \sqrt 3$ unit cell of WSe_2_ and 2 × 2 unit cell of Au (111) faces were constructed to match with each other. As the properties of WSe_2_ are hypersensitive to strain, we adjusted the Au lattice parameter to be commensurable to that of WSe_2_. The strains applied on Au in all the Au/WSe_2_ interface models are less than 1%. To model the Au surface, we used six layers of Au atoms. Considering that the interface has little impact on the bottom several layers of Au atoms, the bottom three layers of Au atoms were fixed.

### Material characterization and electrical measurement

The electrical characteristic measurements were characterized in a Lakeshore PS-100 cryogenic probe station at room temperature in vacuum, using Keysight B2900A source measurement unit (SMU). Besides, for the CMOS logic functions, the voltage transfer characteristics were measured using an Agilent B1500A Semiconductor Parameter Analyzer.

## Supplementary information

Supplementary Information

## Data Availability

The data that support the findings of this study are available from the corresponding author upon reasonable request.
