# Supplementary material for: Doping-free complementary WSe2 circuit via van der Waals metal integration
Source: Nat Commun. 2020 Apr 20;11:1866. doi: 10.1038/s41467-020-15776-x (PMC7171173; doi:10.1038/s41467-020-15776-x)
Supplement: Supplementary file 1 — Supplementary Information [file 41467_2020_15776_MOESM1_ESM.pdf]

Supplementary Information for

Doping-free complementary WSe_2 circuit via van der Waals metal integration

Kong et al.

Contents:

Supplementary Figures 1-12
Supplementary Table 1
Supplementary References

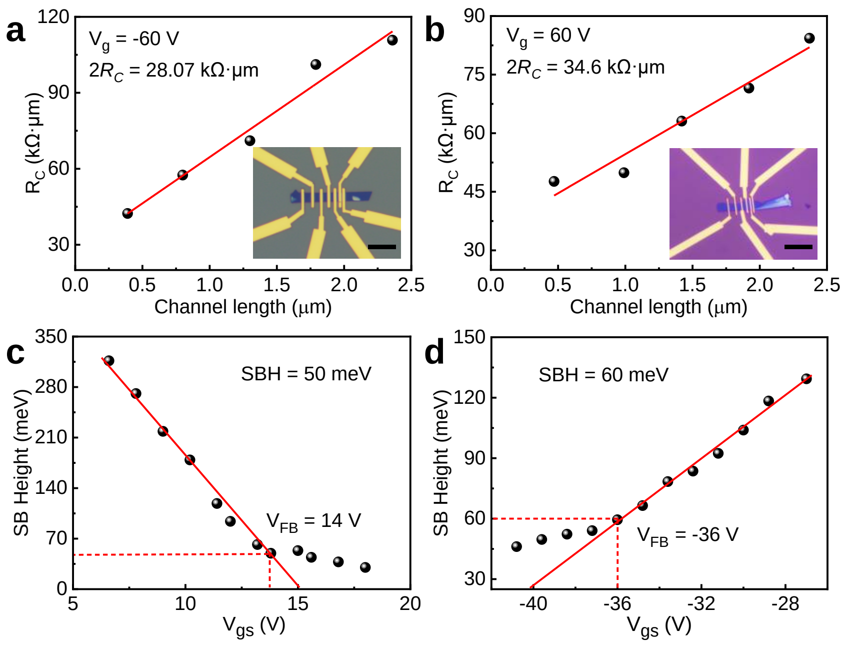

Supplementary Figure 1. Contact resistance (*R_c*) and Schottky barrier height (SBH) with vdW integrated and evaporated Au electrodes. a, R_c extraction of WSe_2 transistors by transfer length method (TLM) (using various channel lengths) with vdW integrated Au electrodes. The R_c of WSe_2 transistors is extracted to be 14 kΩ·μm at V_g = -60 V. Inset: the optical image of WSe_2 transistor of the corresponding device for R_c extraction. Inset scale bar is 10 μm. **b,** Contact resistance extraction of WSe_2 transistors by TLM structure with direct deposited Au contact. The R_c of WSe_2 transistors is extracted to be 17 kΩ·μm at V_g = 60 V. Inset: the optical image of WSe_2 transistor of the corresponding device for R_c extraction. Inset scale bar is 10 μm. **c,** The extracted p-type SBH (for device with vdW integrated Au contact) by temperature dependent measurement, where the flat-band hole barrier is 50 meV, as indicated by the red dash line. **d,** The extracted n-type SBH for device with direct deposited Au contact, where the flat-band electron barrier is 60 meV, as indicated by the red dash line. The bias voltage for SBH extraction (in panel **c**, **d**) is fixed at 100 mV.

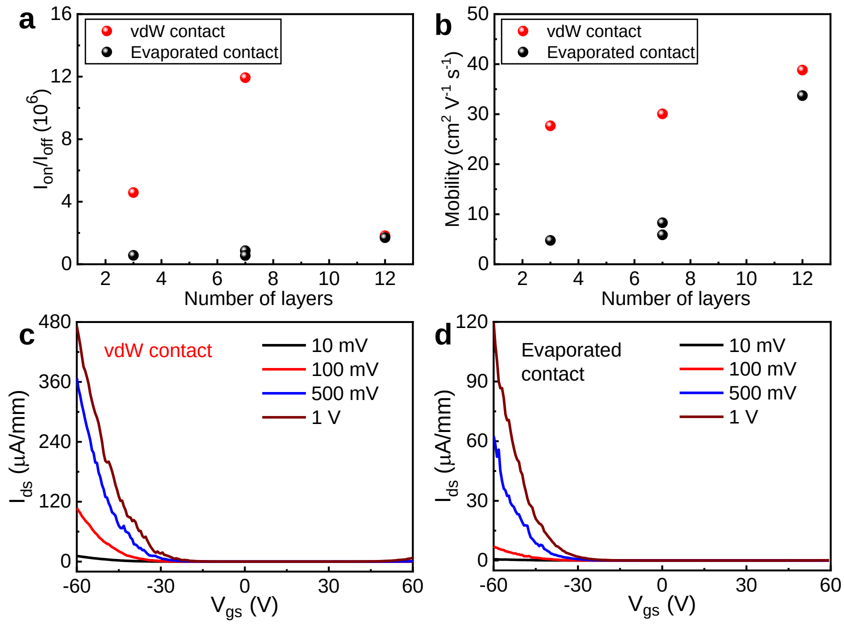

Supplementary Figure 2. The electrical characterization of WSe_2 transistors and monolayer WSe_2 device measurement. a. I_{on}/I_{off} of WSe_2 transistors with different layers (3 layers, 7 layers, 12 layers) using both the vdW integrated and conventional evaporated devices. **b.** Mobility of WSe_2 transistors with different layers (3 layers, 7 layers, 12 layers) using both the vdW integrated and conventional evaporated devices. **c,** I_{ds}-V_{gs} transfer curves of monolayer WSe_2 transistor by using vdW Au electrodes. **d**. I_{ds}-V_{gs} transfer curves of monolayer WSe_2 transistor by using conventional deposited Au electrodes. As shown in (**c**, **d**), p-type transfer characteristic is consistently observed for monolayer devices using both integration methods (vdW integration and direct deposition) of Au contacts, consistent with trend in Fig. 2g.

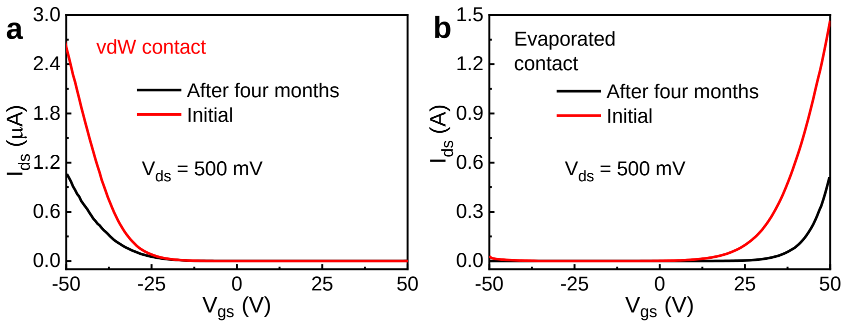

Supplementary Figure 3. Device stability for both contact approaches. a, I_{ds}-V_{gs} transfer curves of the initial (red curve) and after four months of storage (black curve) at room temperature in ambient atmosphere of a WSe_2 device contacted with vdW Au electrodes. **b,** I_{ds}-V_{gs} transfer curves of the initial (red curve) and after four months of storage (black curve) at room temperature in ambient atmosphere of a WSe_2 device contacted with conventional deposited Au electrodes.

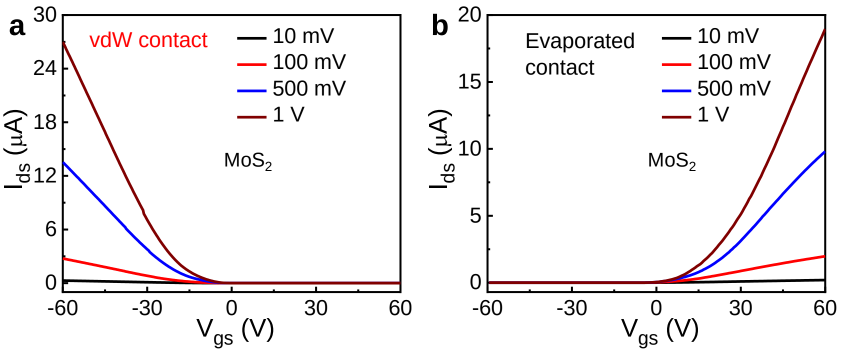

Supplementary Figure 4. Transfer characteristics of MoS_2 transistors with vdW integrated and evaporated Pt electrodes. a, I_{ds}-V_{gs} transfer curves of MoS_2 transistor using vdW Pt electrodes, where p-type device behavior is observed. **b,** I_{ds}-V_{gs} transfer curves of MoS_2 transistor using conventional deposited Pt electrodes with n-type device characteristic. The V_{ds} bias voltage is 0.01 V (black), 0.1 V (red), 0.5 V (blue), and 1 V (brown).

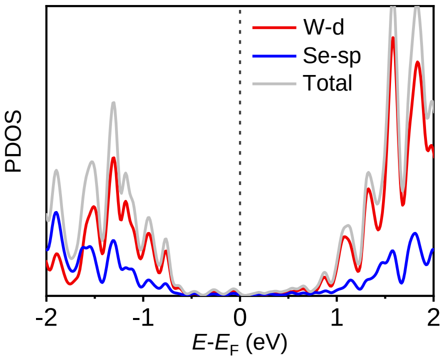

Supplementary Figure 5. Partial density of states (PDOS) of WSe_2 in non-close-contact of Au and monolayer WSe_2. The gray, red, blue lines represent the total DOS of WSe_2, d-orbitals of W atoms, s, p-orbitals of Se atoms, respectively.

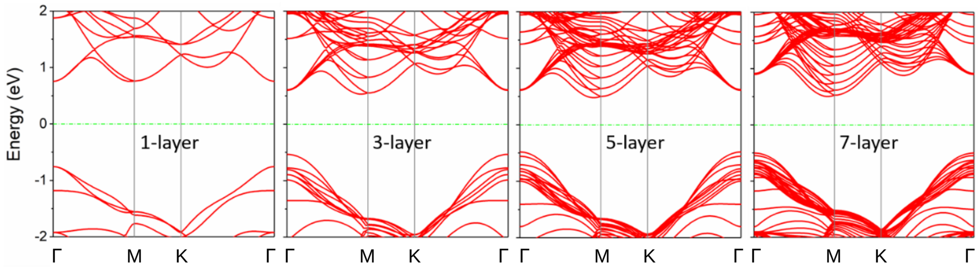

Supplementary Figure 6. Calculated band structures of free-standing WSe_2 with different layers.

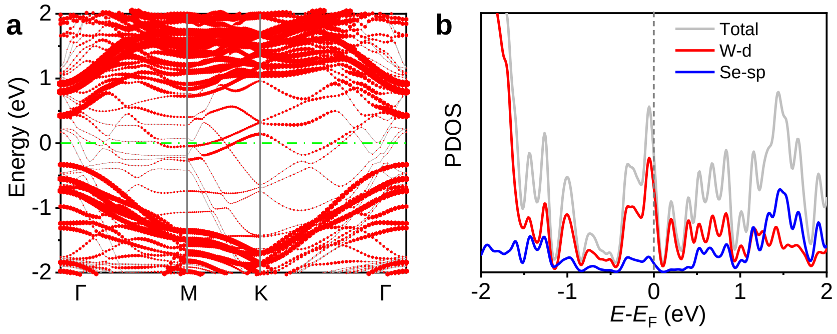

Supplementary Figure 7. Electrical properties of interfacial system for close-contact model. a, Calculated band structure of interfacial system for close-contact model. Red dots represent the projected band structure of WSe_2. **b,** PDOS of WSe_2 in close-contact of Au and monolayer WSe_2. The gray, red, blue lines represent the total DOS of WSe_2, d-orbitals of W atoms, s, p-orbitals of Se atoms, respectively.

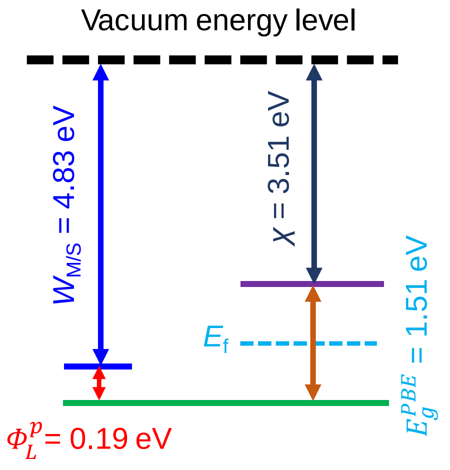

Supplementary Figure 8. The band structure of composite electrode (Au and the underlying WSe_2) and the free-standing monolayer WSe_2. The blue line represents the Fermi level of composite electrode, and the light blue dash line denotes the Fermi level of free-standing WSe_2. The purple line and green line represent the CBM and VBM of free-standing WSe_2, respectively.

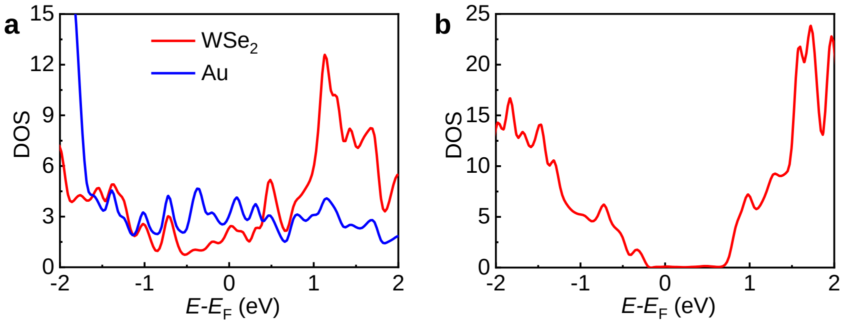

Supplementary Figure 9. DOS of interfacial system in close-contact model of Au and multilayer WSe_2 system. a, DOS of Au and the underlying first layer WSe_2 in close-contact model. **b,** DOS of the rest WSe_2 underlying the first layer WSe_2 in close-contact of Au and multilayer WSe_2 system.

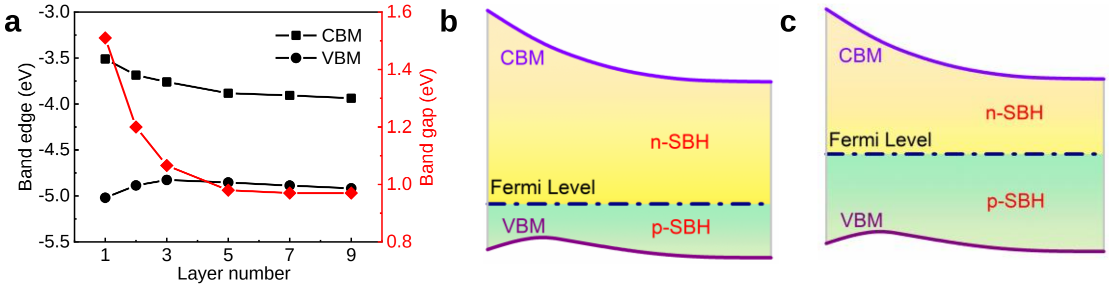

Supplementary Figure 10. Variation of band edges and schematic diagram of SBH variation with WSe_2 layer number. a, Variation of band edges and band gap of pure WSe_2 with layer number. **b,** Schematic diagram of SBH variation with layer number for non-close-contact. The variation of band edges of WSe_2 with layer number mainly contributes to the variation of SBHs. **c,** Schematic diagram of SBH variation with layer number for close-contact model. The polarity transition (from p-type to n-type) and the band alignment could be majorly attributed to the change of WSe_2 band edges with increasing thickness, as well as the smaller metallized WSe_2 work function of 4.83 eV (compared to 5.24 eV of Au in vdW contact).

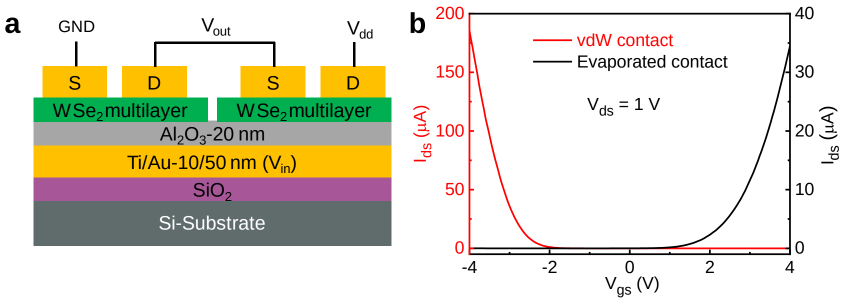

Supplementary Figure 11. Schematic cross-sectional view of inverter and transfer characteristics with different contact approaches. a, Schematic cross-sectional view of complementary inverter. **b,** Transfer characteristic curves of p-type and n-type in linear scale of WSe_2 transistors with the bias voltage of 1 V on Al_2O_3 dielectric.

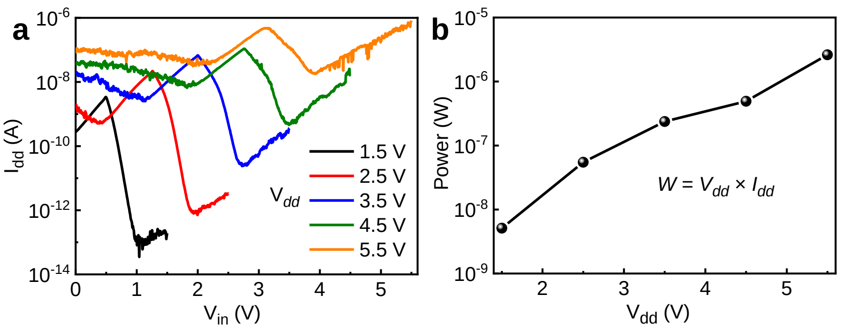

Supplementary Figure 12. The static current（I_{dd}）of inverter and the static peak energy consumption as a function of the input voltage (V_{in}). a, The corresponding static current I_{dd} curves of WSe_2 inverter as a function of input voltage with different V_{dd}. **b,** the static peak energy consumption of the inverter as a function of V_{dd} from 1.5 V to 5.5 V.

Table 1. Comparison of TMDs-based inverter with previous literatures.

Material	V_{dd} (V)	Gain	Reference
BP-MoS_2	3	152	1
WSe_2-$MoSe_2$	3	23	2
BP	2	46	3
	3	2	4
WSe_2-MoS_2	5	27	5
	3	10	6
MoS_2	5	60	7
	2	38	8
	3	22	9
$MoTe_2$	3	195	10
	3	98	11
	2	9.2	12
	20	1.5	13
WSe_2	8	40	14
	5	25	15
	3	12	16
	5.5	340	This work

Supplementary Reference

1. Huang M. et al. Multifunctional high-performance van der Waals heterostructures. *Nat. Nanotechnol.* **12**, 1148−1154 (2017).
2. Chiu M. H. et al. Metal-guided selective growth of 2D materials: demonstration of a bottom-up CMOS inverter. *Adv. Mater.* **31**, e1900861 (2019).
3. Koenig, S. P. et al. Electron doping of ultrathin black phosphorus with Cu adatoms. *Nano Lett.* **16**, 2145−2151 (2016).
4. Liu Y. & Ang K.-W. Monolithically integrated flexible black phosphorus complementary inverter circuits. *ACS Nano* **11**, 7416−7423 (2017).
5. Jeon P. J. et al. Low power consumption complementary inverters with n-MoS_2 and p-WSe_2 dichalcogenide nanosheets on glass for logic and light-emitting diode circuits. *ACS Appl. Mater. Interfaces* **7**, 22333−22340 (2015).
6. Sachid A. B. et al. Monolithic 3D CMOS using layered semiconductors. *Adv. Mater.* **28**, 2547−2554 (2016).

7. Wachter S., Polyushkin D. K., Bethge O. & Mueller T. A microprocessor based on a two-dimensional semiconductor. *Nat. Commun.* **8**, 14948 (2017).
8. Yu, L. et al. Design, modeling, and fabrication of chemical vapor deposition grown MoS_2 circuits with e-mode FETs for large-area electronics. *Nano Lett.* **16**, 6349−6356 (2016).
9. Lan Y.-W. et al. Scalable fabrication of a complementary logic inverter based on MoS_2 fin-shaped field effect transistors. *Nanoscale Horiz.* **4**, 683−688 (2019).
10. Qi D. et al. Continuously tuning electronic properties of few-layer molybdenum ditelluride with in situ aluminum modification toward ultrahigh gain complementary inverters. *ACS Nano* **13**, 9464−9472 (2019).
11. Liu T. et al. Nonvolatile and programmable photodoping in $MoTe_2$ for photoresist-free complementary electronic devices. *Adv. Mater.* **30**, e1804470 (2018).
12. Park Y. J., Katiyar A. K., Hoang A. T. & Ahn J. H. Controllable P- and N-type conversion of $MoTe_2$ via oxide interfacial layer for logic circuits. *Small* **15**, e1901772 (2019).
13. Lin Y.-F. et al. Ambipolar $MoTe_2$ transistors and their applications in logic circuits. *Adv. Mater.* **26**, 3263−3269 (2014).
14. Kang W.-M. et al. High-gain complementary metal-oxide-semiconductor inverter based on multi-layer WSe_2 field effect transistors without doping. *Semicond. Sci. Tech.* **31**, 105001 (2016).
15. Saptarshi D., Madan D. & Roelofs A. High gain, low noise, fully complementary logic inverter based on bi-layer WSe_2 field effect transistors. *Appl. Phys. Lett.* **105**, 083511 (2014).
16. Tosun M. et al. High-gain inverters based on WSe_2 complementary field-effect transistors. *ACS Nano* **8**, 4948−4953 (2014).
